# Wetting Kinetics and Microstructure Analysis of BNi2 Filler Metal over Selective Laser Melted Ti-6Al-4V Substrate

**DOI:** 10.3390/ma13204666

**Published:** 2020-10-20

**Authors:** Jiankun Liu, Guanpeng Liu, Hua Ouyang, Yulong Li, Ming Yan, Michael Pecht

**Affiliations:** 1 Key Lab for Robot & Welding Automation of Jiangxi Province, Mechanical & Electrical Engineering School, Nanchang University, Nanchang 330031, China; 18846440731@163.com (J.L.); guanpengliu@email.ncu.edu.cn (G.L.); 2 The Engineering Training Center of Nanchang University, Mechanical & Electrical Engineering School, Nanchang University, Nanchang 330031, China; ouyanghua@ncu.edu.cn; 3 Department of Materials Science and Engineering, and Shenzhen Key Laboratory for Additive Manufacturing of High-Performance Materials, Southern University of Science and Technology, Shenzhen 518055, China; 4 Center for Advanced Life Cycle Engineering (CALCE), University of Maryland, College Park, MD 20742, USA; pecht@umd.edu

**Keywords:** wetting kinetics, selective laser melting, BNi2, SLMed TC4, in situ observation

## Abstract

The wetting kinetics of nickel-based filler metal (BNi2) over selective laser-melted Ti-6Al-4V (SLMed TC4) titanium alloy in a protective argon atmosphere is experimentally investigated using a real-time in situ hot stage equipped with an optical microscope. The spreading processes at different temperatures are similar, and the overall wetting/spreading process can be roughly divided into three stages: (i) an initial stage, (ii) a rapid spreading stage, and (iii) an asymptotic stage. Moreover, the wetting kinetics of the BNi2/SLMed TC4 system can be expressed by empirical power exponential function *R^n^~t* with *n* = ~1. In the process of spreading, Ti-based solid solution (Ti(ss)) and intermetallic compound (Ti_2_Ni and TiB_2_) were formed at the interface within the reaction domain, and the phase transition of α’ martensitic to α-Ti and β-Ti also took place. The influence of elevated temperature on the spreading and wetting kinetics of the BNi2/SLMed TC4 system was studied, and the results show that the increase of temperature has a slightly promoting effect on the spreading, but a limited impact on the value of *n*. In addition, the spreading and wetting kinetics of BNi2/SLMed TC4 system are similar to those of BNi2 on conventional forged TC4 substrate.

## 1. Introduction

Numerous papers have studied the wetting behavior of liquid metals over solid substrates at room or elevated temperatures. As a result, Washburn [1] proposed the classic Washburn-type kinetics model, which can be applied in most cases to non-reactive wetting on rough/structured surfaces. However, as for reactive wetting, it is always accompanied by apparent chemical reactions, diffusion, the interaction of fluid flow, and phase transformations [2,3]. Due to the complexity of interface interactions around the triple line, it is not easy to establish a model to reliably and comprehensively elucidate the driving force and dissipation mechanism in the reactive wetting process. Nevertheless, to some extent, experiments or an empirical model can still guide the actual brazing process, which is essential for industrial applications, especially when new brazing materials and techniques are developed.

In recent years, the selective laser melting (SLM) technique has received increasing attention for its potential to manufacture complex-shaped metal parts economically in a short cycle [4]. Accordingly, the mechanical properties [5], corrosion properties [6], fatigue properties [7], fracture toughness [8], and other properties of selective laser-melted (SLMed) materials have been extensively studied. Ti-6Al-4V (TC4) is one of the most popular materials used in SLM owing to its attractive application potential in the aerospace industry and biomedical engineering [9]. Due to the rapid cooling rate in the SLM process, SLMed TC4 is mainly composed of α′ martensite, while conventionally cast TC4 is composed of α and β phases [10]. The presence of fine acicular laths α′ martensite results in the tensile, hardness, and other mechanical indexes of SLMed TC4 titanium alloy being close to or even beyond the traditional castings [11]. However, Yu et al. [12] stated that one of the main reasons for limiting the application of SLMed TC4 is the limited chamber volume of the SLM equipment. Even though the size of the equipment is increasing with the development of the SLM technique, the sizes of the formed parts make it difficult to meet the market demands for large parts [13]. As a consequence, it is necessary to join smaller SLMed components to form larger parts. Conventional bonding processes may be considered to be possible methods to overcome this problem by combining small SLMed parts into larger final parts.

A lot of research has focused on the bonding ability of SLMed parts or materials. Prashanth et al. [14] used friction welding to join Al-12Si parts produced by SLM. They found that significant microstructure variations occurred in the welded zone and ductility increased remarkably. In another paper, Prashanth et al. [15] successfully used solid-state welding processes to join SLMed Ti-6Al-4V components and improve the ductility of SLMed components. Nahmany et al. [16] investigated electron beam autogenous welded SLMed Al-Si-10Mg samples, showing for the first time the feasibility of using the electron-beam welding (EBW) technique on SLMed specimens. Tillmann et al. [17] focused on the process of vacuum brazing conventionally manufactured AISI 316L stainless steel to its non-hipped SLMed counterpart. They evaluated the influence of oxide inclusions and porosity within SLMed microstructures. Xia et al. [13] successfully vacuum brazed 3D printed Inconel 718 alloys with BNi2 amorphous filler metal. Yu et al. [12] demonstrated the ability of laser welding for successfully joining SLMed TC4 to SLMed TC4 and SLMed TC4 to wrought TC4. They illustrated that SLMed TC4 after relieving stress had a good laser weldability. Among all the joining methods, brazing is one of the most commonly used for dissimilar material assembly, because that the brazing process is convenient and the welded joints are of good quality, where the molten liquid metal plays a vital role in the wetting behavior of the solid substrate [18,19]. As a result, in order to control the brazing process and improve joint quality, it is necessary to quantitatively model and predict the wetting phenomenon of the liquid metals over the solid substrate.

Up to now, many studies have centered around the wetting behavior of liquid metals on conventionally cast or forged state TC4 substrates. However, as mentioned above, the microstructure of cast or forged TC4 is different from that of SLMed TC4, and its performance is necessarily different. As a result, there is a need to understand the wettability of liquid filler metal on SLMed TC4, which can provide effective information about brazing techniques and improving the quality of brazing products. On the other hand, the interface reaction depends not only on the composition of the substrate but also on the filler metal. Currently, the brazing of most filler metal and titanium alloys with a melting point below the TC4 phase-transition temperature has been widely studied. In previous work, Yu et al. [20] and Liu et al. [21] studied the wetting behavior of AgCu and AgCuTi filler metal on conventional TC4 substrates under argon atmosphere, respectively. Additionally, in the AgCuTi/TiAl reaction system, Li et al. [22] revealed that the influence on the wetting kinetics is mainly controlled by the dominant chemical reaction in the triple line area, and the substrate surface roughness may have a minimal effect on it. However, the brazing of filler metal and titanium alloys above the phase-transition temperature of TC4 has rarely been studied.

In this study, we specifically selected BNi2, a nickel-based filler with a melting point higher than TC4′s phase-transition temperature, and experimentally investigated the wetting kinetics of it spreading on the SLMed TC4 in an argon atmosphere. The main focus was to explore the impact of high temperature on the wetting kinetics and the microstructure of the SLMed TC4 substrate during the process.

## 2. Materials and Methods

**Figure 1:**
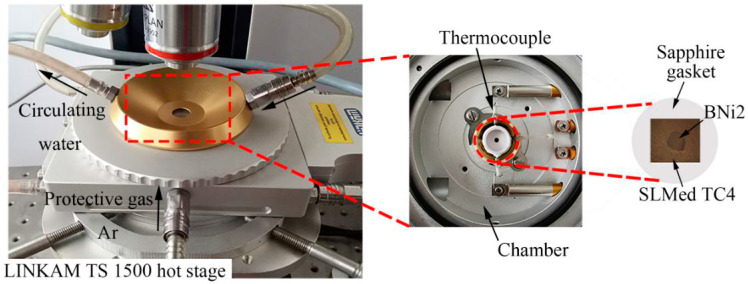
The experimental equipment.

A hot stage microscope was used to monitor the wetting phenomenon of molten BNi2 filler metal over the SLMed TC4 substrate in real time in situ. A SLMed TC4 substrate with dimensions of 5 mm × 5 mm × 2 mm was placed in the chamber of the hot stage (TS 1500, Linkam Scientific Instruments Ltd., Tadworth, UK) installed on an optical microscope (Olympus BX51M, Tokyo, Japan). The parameters of selective laser remelting in the manufacturing process of SLMed TC4 are shown in Table 1. The experimental equipment is shown in Figure 1. Please note that the TC4 substrate is a SLMed sample, and the surfaces were ground step by step using SiC sandpaper in the order of 500#, 800#, 1000#. The BNi2 amorphous foil was made into a disc with a size of ~1.00 mm × 0.05 mm, and then placed in the center of the substrate. The thermomechanical properties of BNi2 were determined via differential scanning calorimetry (DSC, STA449F5, Netzsch, Germany). Before the experiments, the substrates and filler metals were ultrasonically cleaned in acetone, rinsed with distilled water, and dried by hot air blast.

**Figure 2:**
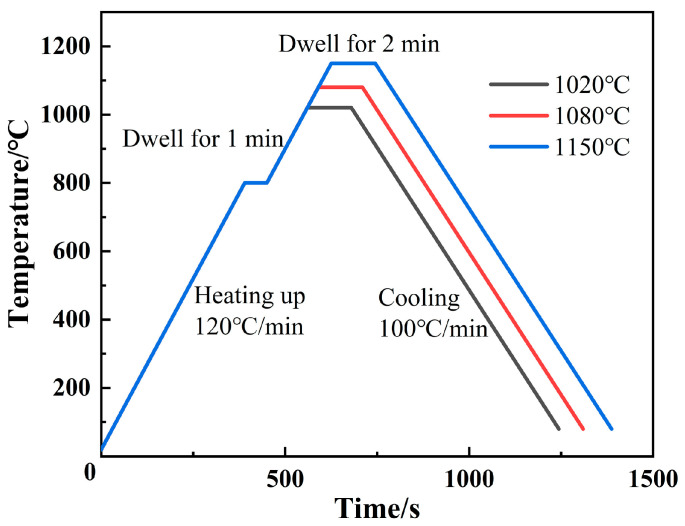
Temperature profile for wetting tests.

All wetting tests were conducted under the protection of ultra-high purity argon (99.999%). Figure 2 shows the thermal cycle curve of the wetting test. The heating cycle was set to heat up with a rate of 120 °C/min, hold for 120 s at the peak temperature (set as 1020 °C, 1080 °C, or 1150 °C) for 120 s, and cool down with a rate of 100 °C/min. To alleviate the temperature hysteresis effect, a dwell time of 1 min at 800 °C was set to make the heating of the filler and substrate evenly. Before starting the heating cycle, the hot-stage chamber was purged with argon for 10 min to ensure that the whole chamber filled with shielding gas. During the heating cycle, a digital camera system with 22 frames per second was used for digital imaging. Please note that at each dwell temperature, the wetting experiments were repeated at least five times to confirm the results. The video started to record as soon as the temperature reached 980 °C, and the movie clips were digitally decomposed into single frames using Virtual Dub 1.10.5 software (originally used by Microsoft Windows), and were correlated with the corresponding temperature and time. The instantaneous equivalent radii of the solid droplets were quantitatively measured by Image-Pro PLUS 6.0 software (Media Cybernetics, Inc.). The calculation steps were as follows: (i) recorded the number of pixels of the scale; (ii) recorded the number of pixels of the spread area; and (iii) calculated the equivalent radius of the instantaneous trajectory of the solid droplet using the following equation:

*R = A*/*B* × (*C*/*π*)^1/2^ (1)

where *R* is the equivalent radius, *A* is the real length of the scale, *B* is the number of pixels in the scale, and *C* is the number of pixels of the spread area.

After the wetting experiment, the wetting samples were cut in the center of the top of the resolidified filler crown by wire cutting. The samples were sealed, ground (successively with 500#, 1000#, 1500#, and 2000# SiC sandpaper), polished (with the 0.5 µm diamond paste) and etched (with the Kroll’s reagent: 100 mL deionized water, 5 mL nitric acid, and 2 mL HF). The cross-section microstructure of the interfacial reaction region of the wetted samples were then investigated by an optical microscope (OM, GX-71 Olympus, Tokyo, Japan) and a scanning electron microscope (SEM, S-3200-N, Hitachi, Japan) with an incidental energy dispersive spectrometer (EDS, IE250X-Max50, Oxford, UK). Additionally, phase analysis was conducted by an X-ray diffractometer (XRD, D8 Advance, Bruker, Germany) and a nano-indenter (iMicro, Nanomechanics, Inc., Oak Ridge, TN, USA) with a Berkovich tip at a maximum depth of 2000 nm.

## 3. Results and Discussion

### 3.1. Filler Metal Characterization

**Figure 3:**
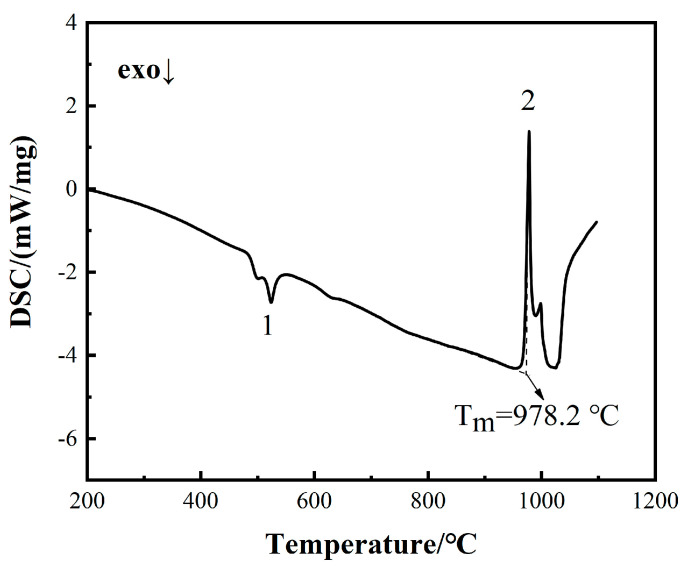
DSC curve of the BNi2 filler metal.

The DSC curve of BNi2 amorphous filler metal is shown in Figure 3. It can be seen that an obviously exothermic peak #1 and an endothermic peak #2 appear. The occurrence of the exothermic peak #1 corresponds to the transition from metastable amorphous produced by the rapid solidification of the foil during processing to a stable crystalline phase, which have been mention in the study of Murray and Corbin [23]. The endothermic peak #2 appearing on the curve corresponds to the melting point of the BNi2 filler metal. Tolkacheva et al. [24] stated that in the DSC curve, the temperature of a thermal event is measured as its onset temperature, more specifically, the intersection point between the tangents to two DSC curve segments, one belonging to the base line and the other to the peak front. Therefore, the melting point of BNi2 is considered to be 978.2 °C, which is consistent with the paper of Arafin et al. [25].

### 3.2. Spreading Phenomena and Data

**Figure 4:**
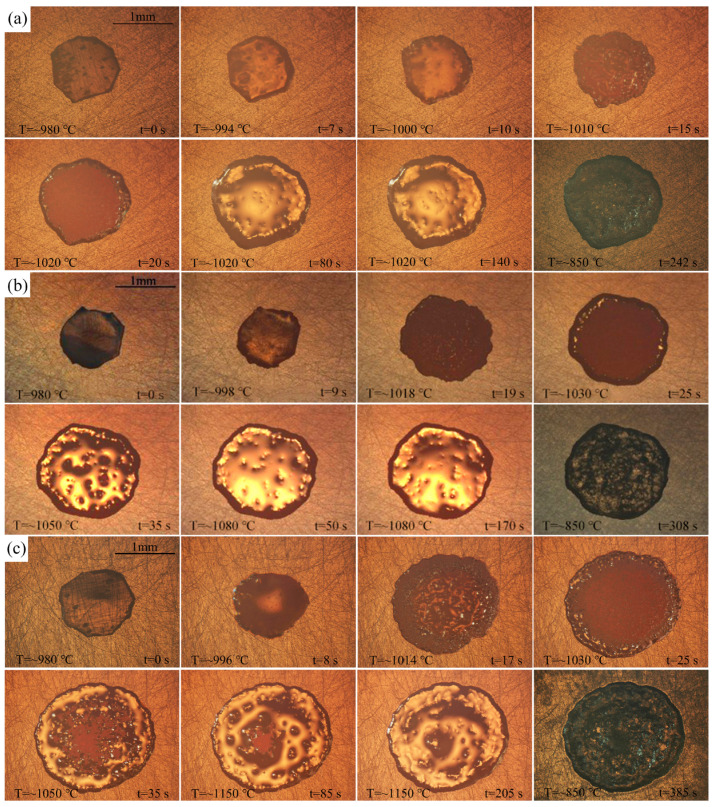
The representative instantaneous images of the wetting process of the BNi2 filler metals over the SLMed TC4 substrates at: (**a**) 1020 °C; (**b**) 1080 °C; and (**c**) 1150 °C.

The effects of temperature on the wetting behavior of BNi2 filler metal over the SLMed TC4 surface were investigated. According to the melting point of the BNi2 alloy, three brazing temperatures of 1020 °C, 1080 °C, and 1150 °C were set for the wetting experiments. The representative instantaneous images draw from the video recorded during the spreading process were shown in Figure 4. As the temperature comes closer to the melting point of BNi2, its size decreases slightly within 1–2 s (e.g., at 994 °C and 7 s). When the temperature increases further, considerable spreading can be observed, until the maximum spreading limit is achieved (e.g., at 1020 °C and 140 s). After cooling, the BNi2 filler metal resolidifies as a residual filler layer over the SLMed TC4 surface. Moreover, by comparing Figure 4a–c, it can be clearly observed that the final spreading area increases with the increase of the brazing temperature.

**Figure 5:**
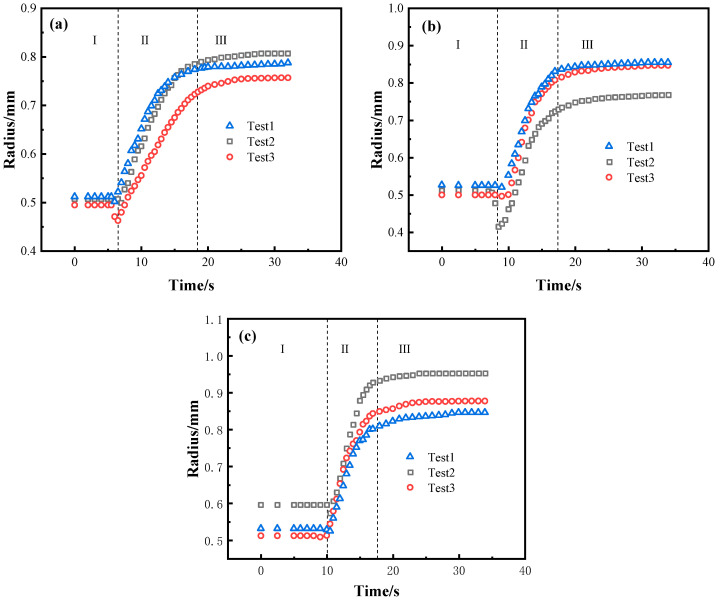
The relationship between the equivalent spreading radius and time of the BNi2/SLMed TC4 wetting system at: (**a**) 1020 °C; (**b**) 1080 °C, and (**c**) 1150 °C.

Figure 5 presents the relationship between the equivalent spreading radius and time of the BNi2/SLMed TC4 wetting system at 1020 °C, 1080 °C, and 1150 °C, respectively. Although the tests are conducted under the same conditions, the results under the same parameters may show a minimal difference due to the related error of the filler size, the substrate morphology, and the heating resistance between the contacting surfaces. It can be seen that the wetting processes of BNi2 brazing filler on SLMed TC4 at three different dwell temperatures are similar: the equivalent spreading radius decreases slightly and then increases rapidly with a large rate and finally approaches an asymptotic value.

For each dwell temperature (e.g., at 1020 °C shown in Figure 5a), the overall spreading process can be divided into three distinct stages (i–iii) as follows:

(i) Initial stage. The BNi2 filler metal melts slowly and contracts rapidly as the temperature comes closer to the melting point of the BNi2. The reason could be that the SLMed TC4 substrate surface can easily react with oxygen and be oxidized during the process of sample preparation and/or heating, which means that the BNi2 brazing filler is actually in direct contact with a surface oxide layer [26]. As a result, near the melting point, the cohesion of the BNi2 filler metal induced by the free energy of the solid/liquid interfacial tension is greater than the adhesion between the solid and liquid, inducing a contact angle larger than 90°. It should be noticed that this process only lasts 1–2 s and then quickly begins to move on to the next stage.

(ii) Rapid spreading stage. The oxides on the surface of SLMed TC4 gradually rupture with the rise of temperature, and the direct contact of the BNi2 molten metal and surface of the TC4 substrate triggers chemical reactions between Ni and Ti. At the same time, the spreading of the filler alloy on the substrate was obviously accelerated. This transition is mainly attributed to the reduction of the solid/liquid interfacial tension resulted by the Ti-Ni phase formed in the reaction between Ti and Ni at the interface and the promotion of free energy released by the interfacial reaction to the spread of the wetting. It is worth noting that the rapid spreading stage covers up to about 90% of the entire spreading area.

(iii) Asymptotic stage. As the reaction between BNi2 filler and the substrate lasts for a period of time, the Ni content in the filler metal further decreases, resulting in suspension of the reaction and showing an asymptotic trend. Ultimately, through the final asymptotic stage, the molten filler reaches the maximum spreading area and terminates. Moreover, compared with the results at 1020 °C and 1080 °C, the wetting and spreading process takes less time when it conducted at 1150 °C. The increase of temperature has a slight promoting effect on the spreading: the higher the temperature, the faster the chemical reaction and the larger the spreading area. Similar conclusions were drawn by Landry et al. in the Al/Cv system [27].

### 3.3. Microstructure Analysis

**Figure 6:**
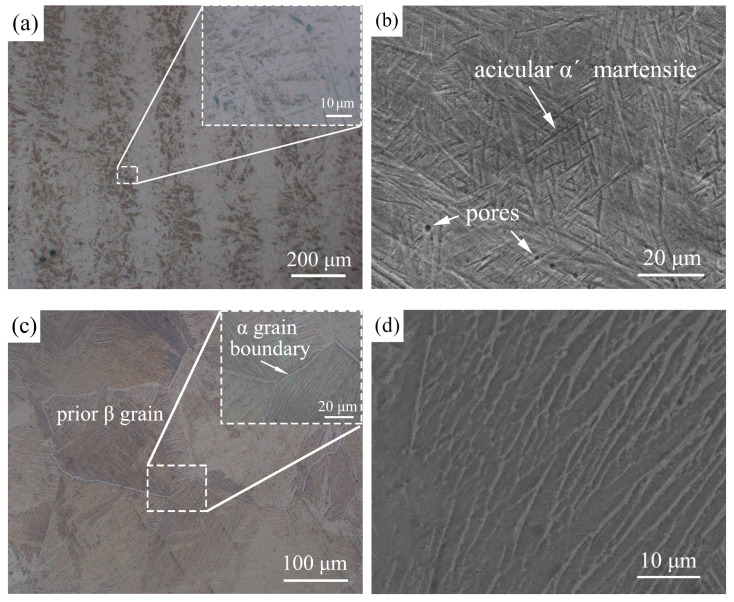
Microstructure of the SLMed TC4: (**a**) OM image and (**b**) SEM image before wetting; (**c**) OM image and (**d**) SEM image after wetting.

Figure 6 presents the typical microstructure of SLMed TC4 alloy before and after wetting, respectively. During the SLM process, TC4 metal powder was heated rapidly by high-energy laser irradiation and melted into a liquid. A molten pool was formed on the substrate, the size of which was equal to the spot size. As the laser beam advances, the liquid alloy in the molten pool cools rapidly and solidifies by convection, conduction, and thermal radiation. The structures in Figure 6a exhibit characteristics of alternating light and dark lines, because of different crystal orientations. Due to the fast cooling rates in the SLM process, the microstructure of SLMed TC4 is mainly composed of acicular α′ martensite phase [28], as shown in Figure 6b. SLMed TC4 consisting entirely of α′ phase generally exhibits a poor elongation at failure, because α′ martensite grains do not form slat colonies with the same orientation, and the effective slip length is limited to a single size [29]. However, the microstructure of SLMed TC4 changed after high temperature wetting with BNi2 filler metal. Coarse prior β grains and α grain boundaries (Widmanstatten structure) can be clearly seen in Figure 6c, and Figure 6d is an enlarged view of the prior β grains. Generally, when TC4 alloy does not deform after being heated above the β phase transition temperature, and the temperature is lowered at a slow cooling rate, a Widmanstatten structure will be formed. In the prior β grains, a few “clusters” with larger sizes are formed, and in the same cluster, there are more α sheets parallel to each other and have a definite orientation relationship [30,31]. The Widmanstatten structure has a high creep strength and fracture toughness, but its plasticity is low, the reason is that the size of the prior β grains is larger than that of other types of structure, and there are continuous grain boundaries [32].

**Figure 7:**
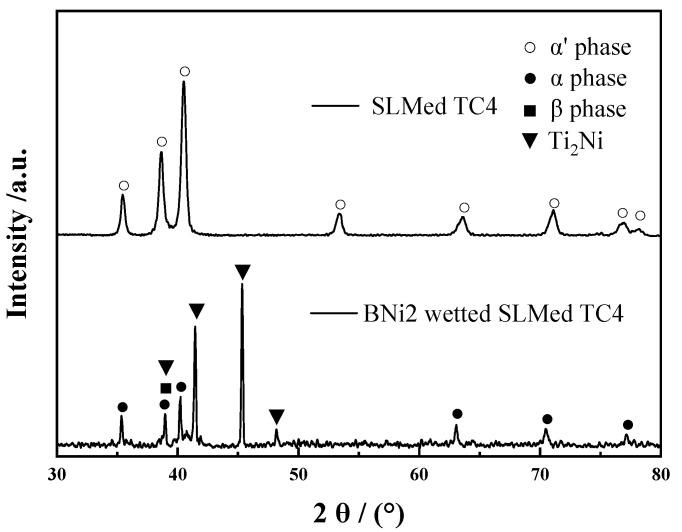
XRD patterns of surfaces of the SLMed TC4 and BNi2 wetted SLMed TC4.

Figure 7 shows XRD patterns of surfaces of the SLMed TC4 and BNi2 wetted SLMed TC4. The XRD patterns of the SLMed TC4 show the presence of α′ martensitic peaks, which is similar to the study of Krakhmalev et al. [33]. However, the α′ transforms to α and β phase after high-temperature wetting of BNi2, and Ti_2_Ni occurs. This also confirms the above organizational analysis.

**Figure 8:**
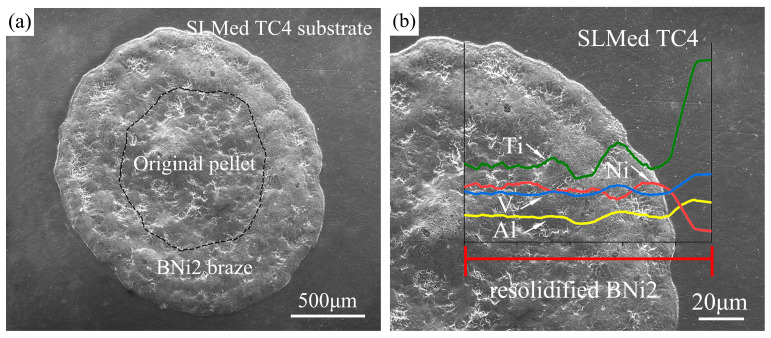
(**a**) A typical top view of the wetted sample; (**b**) the line scan analysis of the main element distribution from the center to the edge of the wetted sample.

**Figure 9:**
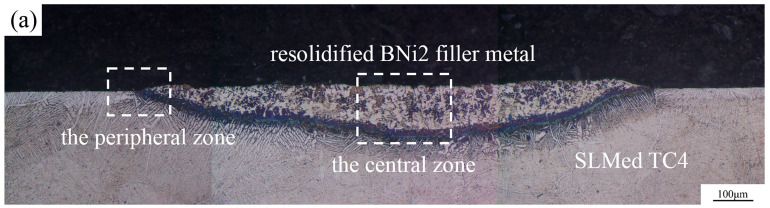

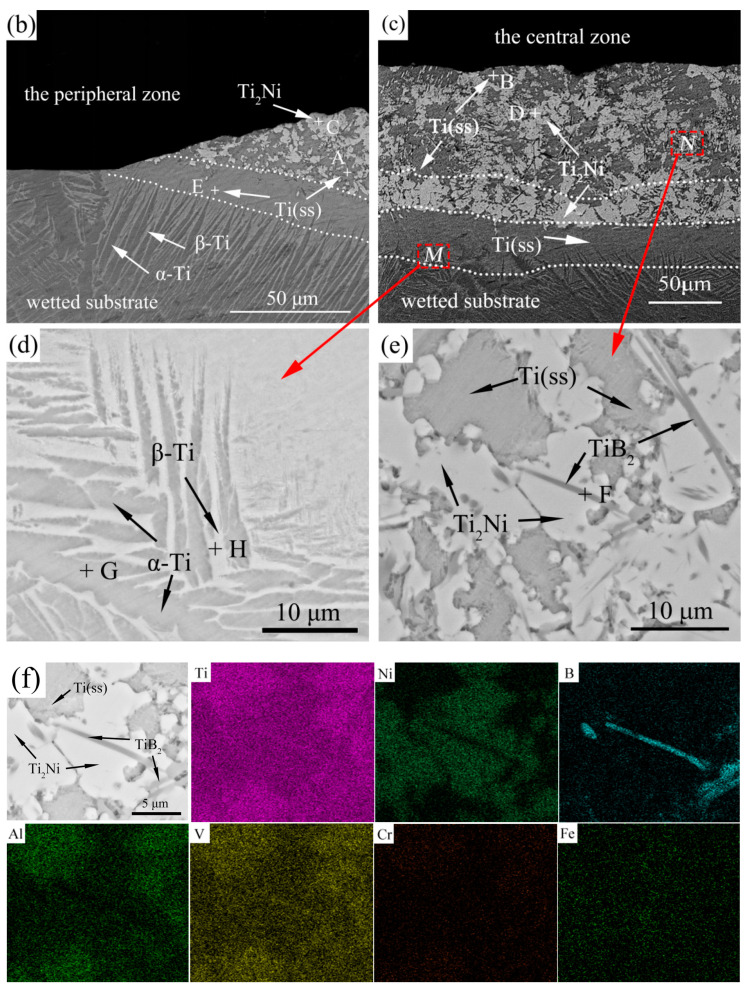
Typical resolidified BNi2 filler metal over the SLMed TC4 with a peak temperature of 1080 °C and dwell time of 120 s: (**a**) the optical micrograph of the whole cross-section; (**b**) the edge area of the cross-section; (**c**) the central area of the cross-section; (**d**) the magnified topography of area M in (**c**); (**e**) the magnified topography of area N in (**c**); and (**f**) EDS mapping of the partial area in Figure 9d.

There are no significant differences in the interfacial microstructures and phase distributions at different temperatures, so only the microstructure of one typical sample is presented for the analysis. Figure 8a shows a typical top view of the wetted sample, where the boundary of the original pellet is marked by black dashed lines. It can be observed that the filler metal spreads outward, forming a nearly circular resolidified residual filler. A line scan analysis was conducted from the filler center to the substrate edge along the radial direction. Figure 8b shows that the content of several main elements (Ti, Al, V, Ni) varies from the center to the edge of the resolidified BNi2 filler metal. During the spreading process, the substrate dissolved a little bit into the liquid filler metal, and interdiffusion and chemical reaction occurred between the substrate and the liquid filler metal, which resulted in the elements distribution changing across the interface. Figure 9a presents the optical micrograph of the whole cross-section obtained by cutting from the middle of the wetting sample. Figure 9b and c show the typical microstructure of the peripheral zone and the central zone of the sample cross-section after wetting, respectively. Figure 9d,e show the magnified images of the marked area M and area N in Figure 9c, respectively. Figure 9f shows the EDS mapping of a local place in Figure 9e. As shown in Table 2, several reaction phases were formed and the chemical compositions of each phase in Figure 9b–e measured by EDS are listed. Sites A and B were mainly composed of Ti and a small amount of Al, V, Ni, Cr, and Fe, and this eutectoid structure was inferred to be a Ti-based solid solution with some other elements, hereinafter referred to as Ti(ss). The EDS results in the light-colored region (e.g., sites C and D) show that the stoichiometric ratio of Ti and Ni was close to 2:1, which was confirmed as Ti_2_Ni by the previous XRD results (shown in Figure 7). Site F of the acicular structure showed in Figure 9d were primarily comprised of B and Ti in a close ratio of 2:1, which was considered to be TiB_2_. The previous XRD results did not show the presence of TiB_2_ because that its content is too few to be detected. The presence of a small amount of acicular TiB_2_ will play a fixed role and increase the interfacial strength. On the other hand, α-Ti and β-Ti can be observed at the edge domain, which means that the α′ phase changed to the α phase and partially further transformed into β-Ti during the high-temperature wetting. This result is reasonable because the wetting temperature is obviously higher than the transforming temperature of α-Ti to β-Ti in TC4 [34]. Nanoindentation tests show the nano hardness of the Ti_2_Ni phase was 8.89 ± 0.2 GPa, while the SLMed TC4 substrate before and after wetting were 6.61 ± 0.25 GPa and 5.18 ± 0.2 GPa, respectively. Layered hard and brittle intermetallics should normally be avoided during brazing; fortunately, the Ti_2_Ni phases are not dispersed in a layer-like structure. Also, the transformation of the α’ phase to the α and β mixed phases indicates a hardness decrease in the substrate, which may induce a soft toughening effect for the SLMed TC4.

### 3.4. Wetting Kinetics

Based on the combination of liquid and solid, wetting processes can be divided into two types: non-reactive wetting and reactive wetting. In most cases, non-reactive wetting systems follow a Washburn-type relationship, which indicates that the wetting spreading radius has a relationship of power n with time, namely, *R^n^~t*, *n* = 10. Tanner [35] revealed that the propagation time of the non-reactive system was only several milliseconds. As for reactive wetting systems, Eustathopoulos [36] declared that the propagation time was about 10^1^–10^4^ s, which is several orders of magnitude higher than that of non-reactive wetting systems. This indicates that the diffusion rate of a reactive wetting system is primarily determined by chemical reactions, while non-reactive wetting systems are determined by viscous dissipation. In this study, the reaction time was around 10 s and the BNi2 filler depleted quickly, which may be because the reaction was too intensive at the high temperature of around 1000 °C. Similarly, the reactive wetting system can also be described by the Washburn-type formula *R^n^~t* with different values of *n*. According to previous studies, Tillmann et al. [37] declared that when the diffusion rate of an element is higher than the triple-line reaction rate, the wetting kinetics are reaction-limited, where *n* = 1; while when the diffusion rate of active element is less than the reaction rate, Landry et al. [27] and Mortensen et al. [38] concluded that diffusion is the dominant rate-limiting factor, where *n* = 4.

**Figure 10:**
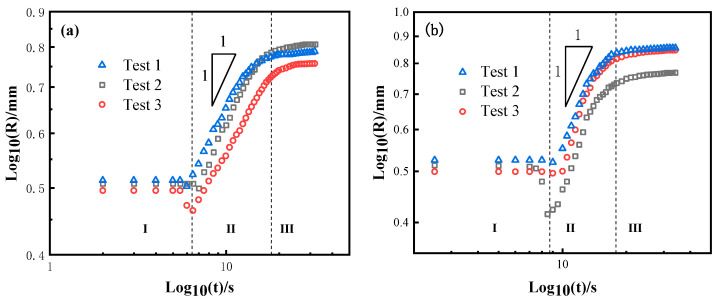

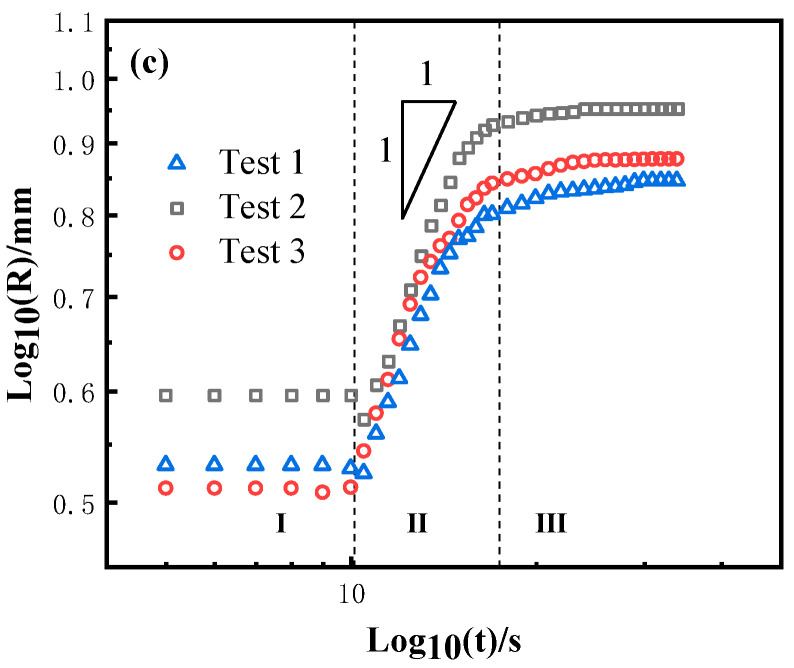
The logarithmic relationship between equivalent expansion radius and time of the BNi2/SLMed TC4 system at: (**a**) 1020 °C; (**b**) 1080 °C, and (**c**) 1150 °C.

For better understanding of the wetting mechanism, as shown in Figure 10, the logarithmic relationship between the equivalent expansion radius and time of the BNi2/SLMed TC4 system was plotted. As shown in Figure 10a–c, at 1020 °C, 1080 °C, and 1150 °C, the spreading of the BNi2 molten filler metal on SLMed TC4 substrates on the rapid spreading stage follows the relation *R^n^~t* with *n* = ~1. Hence, it can be deduced that the rapid spreading stage (*n* = ~1) is dominated by chemical reactions. Please note that the same *R^n^~t* (*n* = ~1) relation for the three high temperatures in this study indicates that in the BNi2/SLMed TC4 system, a slight temperature change may have a limited impact on the value of *n*. Landry and Eustathopoulos [39] came to a similar conclusion that the spreading of the molten liquid filler metal was controlled by chemical reactions in the study of pure aluminum/vitreous carbon system, and similar spreading behavior was observed at temperatures of 1100 K and 1190 K, which is consistent with our experimental results. Similarly, in the Sn-35Bi-1Ag/Cu system, at 483 K and 533 K, Li et al. [40] found that the values of *n* for the corresponding spreading stage is the same, which means the temperature has limited effects on the spreading rate.

**Figure 11:**
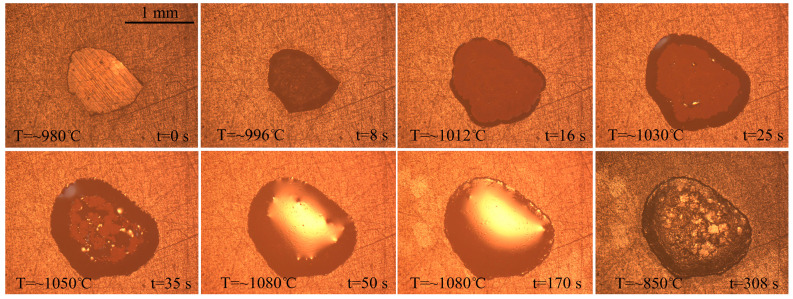
The representative instantaneous images of the spreading process of the BNi2 filler metals over the conventional forged TC4 substrates at 1080 °C.

**Figure 12:**
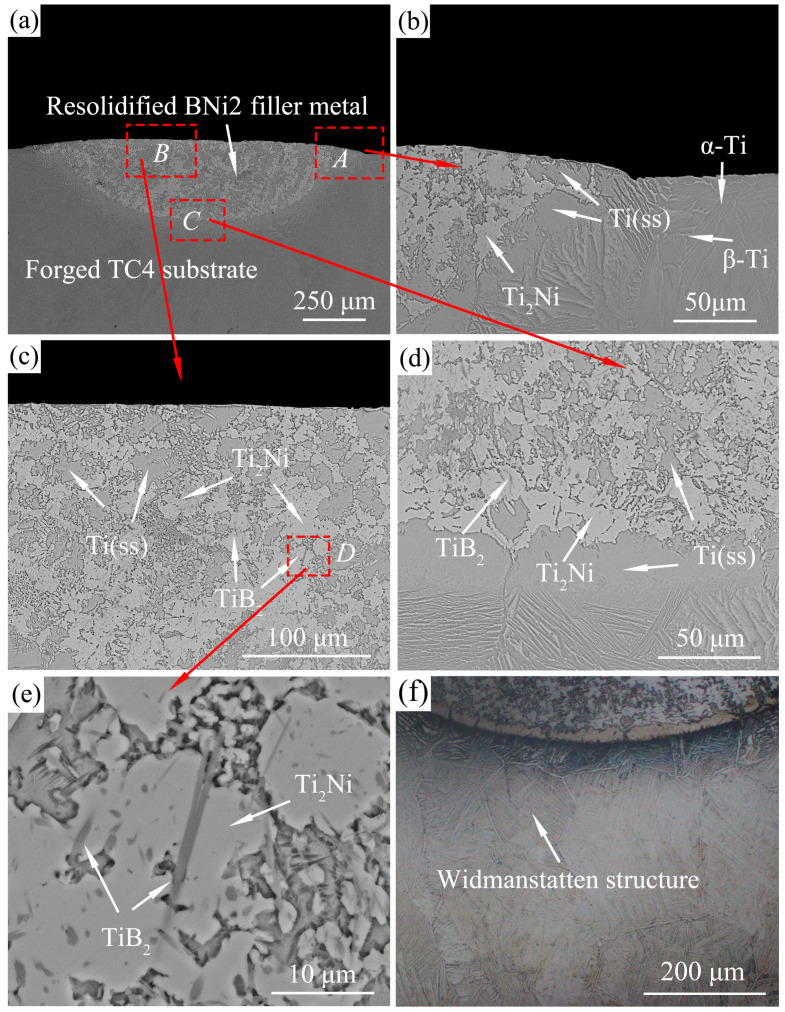
Typical resolidified BNi2 filler metal over the conventional forged TC4 with a peak temperature of 1080 °C and dwell time of 120 s: (**a**) the whole cross-section; (**b**) the edge area of the cross-section; (**c**) the central area of the cross-section; (**d**) the bottom interface area of the cross-section; (**e**) the magnified topography of area *D* in (**c**); and (**f**) the optical micrograph of the substrate after wetting.

**Figure 13:**
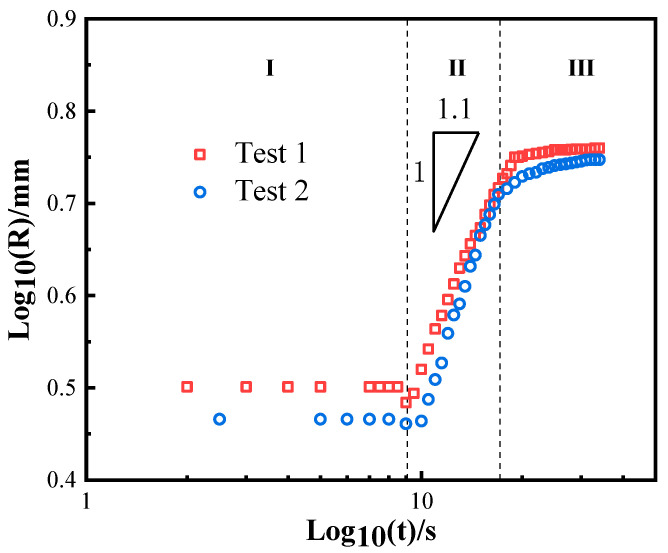
The logarithmic relationship between the equivalent expansion radius and time of the BNi2/TC4 system at 1080 °C.

For comparison, wetting experiments of BNi2 filler metal on conventional forged TC4 were carried out under the same conditions. The representative instantaneous images of the spreading process of the BNi2 filler metals over the conventional forged TC4 substrates at 1080 °C are shown in Figure 11. The wetting morphology of BNi2 on forged TC4 is similar to that of BNi2/SLMed TC4. Figure 12 demonstrates different areas of the typical resolidified BNi2 filler metal on the surface of the conventional forged TC4 with a peak temperature of 1080 °C and a dwell time of 120 s. It can be clearly seen that the reaction interface is composed of a large amount of Ti_2_Ni and Ti(ss), and there are also acicular TiB_2_ inclusions, where the reaction products are consistent with that of the BNi2/SLMed TC4 system. Similar to the SLMed TC4 substrates, Widmanstatten structure appeared in the forged TC4 substrates after wetting, as shown in Figure 12f. Furthermore, as shown in Figure 13, a logarithmic relationship between the equivalent expansion radius and time of the BNi2/TC4 system was acquired, whereby the spreading process could also be divided into three stages. The spreading of the BNi2 molten filler metal on conventional forged TC4 substrates on the rapid spreading stage meets the relation of *R^n^~t* with *n* = ~1.1, which is similar to that of the BNi2/SLMed TC4 system (*n* = ~1). It is well understood that although the phases in the two substrates are different (SLMed TC4 is mainly composed of α′, but forged TC4 is mainly composed of α + β) before wetting, the composition of the substrate (both are Ti-6Al-4V) and phases after high temperature wetting are similar, which leads to the similar reactions and wetting kinetics between the filler metal and the substrate during the wetting and spreading process.

## 4. Conclusions

This paper presents an experimental investigation of BNi2 amorphous filler metals over SLMed TC4 substrates. The heating process transformed the SLMed TC4 substrate from α’ phase to α and β phases, and Ti_2_Ni and TiB_2_ were formed in the reaction area. The purpose was to study the influence of elevated temperature on the wetting behavior of the BNi2/SLMed TC4 system. The experiment consisted of brazing at three different temperatures in a controlled atmosphere of high-purity argon. As expected, the higher the temperature, the faster the chemical reaction, and the shorter the spreading process. Of special interest was the wetting process, which can be divided into an initial stage, where the filler metal gradually melted and shrunk, a rapid spreading stage, where the spreading speed was obviously accelerated and the spreading area occupied 90% of the total area, and an asymptotic stage, where the reaction gradually suspended and showed an asymptotic tendency. The wetting kinetics of the BNi2/SLMed TC4 system can be expressed by empirical power exponential function *R^n^~t* with *n* = ~1. Similar interfacial reactions and wetting kinetics (*n* = ~1.1) were also found in the BNi2/forged TC4 system. A case study of BNi2/TC4 brazing indicates that an increase of the brazing temperature can speed up the propagating rate and increase the propagating area, but the influence on the value of *n* is limited.

## Figures and Tables

**Table 1:** Selective laser melting parameters.

Process Parameters	Values
Laser energy density (*VED*)	69.5 J·mm^−^^3^
Laser power (*P*)	275 W
Laser scanning speed (*v*)	1100 mm·s^−^^1^
Printing layer thickness (*h*)	0.03 mm
Overlap spacing (*l*)	0.12 mm

**Table 2:** EDS analysis associated with Figure 9b–e.

Sites	Main Elements (at.%)								Possible Phase(s)
	Ti	Al	V	Ni	Cr	Fe	Si	B	
A	73.32	10.86	4.35	7.06	2.40	0.70	1.31	-	Ti(ss)
B	73.95	11.09	4.33	7.15	2.30	0.81	1.37	-	
C	63.25	5.42	-	27.92	1.24	1.21	0.95	-	Ti_2_Ni
D	63.01	5.3	-	28.42	1.29	1.16	0.38	-	
E	79.28	9.84	4.94	5.39	-	0.55	-	-	Ti(ss)
F	36.18	0.55	-	5.11	-	-	-	58.26	TiB_2_
G	75.51	11.16	4.16	6.06	1.38	0.68	1.06	-	α-Ti
H	74.65	11.25	4.30	6.60	1.58	0.63	0.99	-	β-Ti
